# Perspectives of Clinical Researchers on Engagement With Digital Mental Health Interventions: Qualitative Interview Study

**DOI:** 10.2196/89009

**Published:** 2026-08-06

**Authors:** Bruna Oewel, Keertana Nambiar, Elena Agapie, Madhu Reddy

**Affiliations:** 1 Department of Informatics, University of California, Irvine, Donald Bren Hall, Irvine, CA, 92617, United States, (949) 824-7427

**Keywords:** engagement, digital mental health, user experience, human-centered design, interviews

## Abstract

**Background:**

While technology can widen access to mental health treatments, digital mental health interventions (DMHIs) frequently have low engagement and high dropout rates. A better understanding of user engagement with DMHIs can help researchers design technologies that users are more likely to benefit from. However, a major challenge is that the term “engagement” is very broad, not well-understood, and operationalized differently across projects. Few studies have explored how clinical researchers define and operationalize engagement in DMHI research.

**Objective:**

This study investigated how clinical researchers operationalize user engagement with DMHIs in academic settings. Investigating operationalization can help identify gaps and inform strategies to better operationalize engagement in DMHIs according to intervention goals.

**Methods:**

We conducted exploratory qualitative semistructured interviews via Zoom (Zoom Communications, Inc; May 2023 to February 2024) with 12 clinical mental health researchers who had developed DMHIs using human-centered design methods. We recruited participants via purposive and snowball sampling. The interviews focused on understanding what participants considered engagement, how they measured it, the strategies they used to support or increase engagement, and the barriers they faced. We inductively coded the transcripts and conducted thematic analysis, iterating on codes and themes collaboratively.

**Results:**

We identified 3 dimensions of engagement for DMHIs: digital mental health components (ie, intervention, technology, and human support), levels of engagement (micro and macro), and visibility of the engagement (visible and invisible). We also described the challenges of designing DMHIs for engagement. Participants described components as overlapping; some viewed the technology and intervention as one, while others viewed the technology as distinct. Users should experience components as integrated. Within the levels of engagement, clinical researchers focused on designing for macroengagement but primarily measured microengagement through quantitative measures. Participants distinguished the visibility of engagement between what was capturable and measurable (visible engagement) and what could not be captured (invisible engagement). A major barrier to engagement was overcoming the invisibility of opportune moments for users to engage with the DMHI.

**Conclusions:**

Clinical mental health research focuses on end point clinical outcomes, while user engagement refers to the dynamic interaction with DMHIs in real-world settings, encompassing behavioral, cognitive, and affective user involvement. This tension highlights the need to operationalize engagement to better understand how users engage with DMHIs. A mixed method approach to capturing engagement would better align with clinical researchers’ goals of understanding macroengagement. The dimensions of engagement (components, levels, and visibility) extend previous conceptualizations by providing support at the start of designing for the engagement of DMHIs. While capturing invisible aspects can still be challenging, awareness of the visibility of engagement can help researchers align their measurement and evaluation approaches with their macroengagement goals, instead of focusing on aspects of engagement that are readily visible.

## Introduction

### Background

Poor mental health is increasing, affecting 42.5% of US adults, according to a 2022 survey [1]. People face barriers to accessing traditional care, such as a lack of service availability and high costs [2,3]. Digital mental health interventions (DMHIs) have been shown to improve mental health [4-7], with evidence highlighting their potential to help users achieve their mental health goals (eg, symptom reduction or the development of new coping skills) [8,9]. Engagement is considered a key mediator of outcomes [9-12]. However, DMHIs currently experience low engagement and high dropout rates [7,13-15]. Among commercial DMHIs, the median retention rate was 3.9% after 15 days of use [16], indicating a need to both understand and improve user engagement with these tools [14,17]. The challenge of low engagement with DMHIs is further complicated by a lack of consensus on what engagement means and a lack of understanding of how engagement is applied in their design [10,17,18].

Engagement with digital interventions is a multidimensional concept often discussed as being composed of 3 constructs: cognitive (eg, effort and attention), affective (eg, emotions and values), and behavioral (eg, usage and actions) engagement [2,4,19-21]. However, no standard definition of engagement exists across research fields, such as behavioral sciences and human-computer interaction (HCI) [10,18,22]. Existing models of engagement highlight the dynamic process of user engagement with technology that goes beyond discrete technology use [10,23-25]. The foundational model of engagement by O’Brien and Toms [24] presents the temporal stages of engagement, starting with the point of engagement, sustained engagement, end of engagement, and, sometimes, re-engagement. Each stage is characterized by attributes of user experience (eg, positive affect and sensory appeal), based on interviews with users of video games, educational applications, online shopping, and web search [24]. As an extension of this dynamic view, Yardley et al [26] differentiated between the micro- and macro-level engagement. Microengagement, also known as “little e,” refers to discrete or momentary engagement with the digital intervention, particularly with the technology [26,27], including the user interface elements and the behavior change intervention components that are presented in the technology [27]. Macroengagement refers to the user’s engagement with the behavior change process that the DMHI is designed to support [26,27]. Together, these levels capture both discrete and longitudinal contexts of how users engage with digital systems. Our study examined how the micro and macro levels of engagement are applied in the design of DMHIs.

Research on engagement has mainly focused on *conceptualizing and measuring* engagement rather than on understanding how the definitions are reflected in the practice of creating technologies [10,17,18,23,24]. There are engagement models specific to DMHIs, but they tend to focus on measuring or predicting engagement outcomes of a single intervention [28,29]. For example, the Integrative Engagement Model of Digital Psychotherapy [29] combines frameworks from behavioral sciences and HCI to define the phases of engagement with therapist-supported DMHIs. These models advance the understanding of engagement with DMHIs, but do not inform how researchers operationalize engagement. Lipschitz et al [17] emphasized that research needs to advance theories of what drives DMHIs engagement and that this needs to be understood at different levels, in real-world settings, rather than only in clinical trials.

How engagement is measured reflects these conceptual tensions. There is no standard measurement approach for engagement, but quantitative measures are the most commonly used [17,30-32]. Quantitative measures are often used to measure engagement at discrete moments in time [10,25,33] through methods such as log analysis (eg, number of clicks and frequency of views) [10,34]. However, these metrics predominantly capture usage and fail to account for the dynamic process that models describe. There has been an ongoing discussion about expanding beyond traditional quantitative usage metrics, with more mixed methods approaches to measuring engagement (eg, qualitative interviews [10]), to help surface the nuanced and dynamic aspects of engagement [10,23-25].

The challenges of conceptualizing and measuring engagement are amplified by the nature of mental health. Mental health symptoms are dynamic, fluctuating over time [35], and can affect user engagement due to a lack of intrinsic motivation [36] and life barriers [36,37]. Incorporating human support or a human-in-the-loop approach for technical support, accountability, or encouragement is a common strategy to sustain engagement with DMHIs [10,38-40]. Although these strategies tend to enhance engagement, DMHI engagement still remains low, with high dropout rates [7,13,15,41].

### Study Objective

This research focuses on defining engagement constructs and outcome-focused models, but it has not investigated how professionals integrate engagement into the design of DMHIs. Without understanding how engagement is put into practice, even a tested framework might not succeed in improving engagement due to inconsistent applications of engagement measures and strategies [17]. This study aims to expand our understanding of how academic clinical researchers operationalize engagement in DMHIs. To achieve this, we interviewed clinical mental health researchers who created DMHIs for their research projects (ie, pilots and clinical trials). As a first step in understanding how engagement is operationalized in DMHI development, we interviewed clinical researchers because they often lead the development of DMHIs and make decisions about how engagement will be operationalized for these systems. Since little is known about how engagement is operationalized in this context, we used a qualitative interviewing method, as it allowed us to examine in-depth how clinical researchers operationalize engagement. We viewed this approach as particularly useful because the rationales and processes underlying these operationalization decisions are often implicit, nuanced, and context-dependent. This study aims to better understand how clinical researchers operationalize engagement for DMHIs and to discuss strategies to better align these operationalizations with the dynamic nature of user engagement with DMHIs.

## Methods

### Recruitment

We conducted semistructured interviews with 12 clinical mental health researchers based in the United States who develop and evaluate DMHIs. All DMHI projects were part of academic research projects, either as research pilots or as clinical trials. Participants were recruited through a purposive sampling of researchers within the professional networks of the authors. Additional participants were recruited through a snowball strategy from the initial group [42]. We contacted all participants by email, and all researchers contacted agreed to participate. Participants were eligible for the study if they (1) were mental health experts, (2) worked in academia, (3) had directly worked on or supervised digital mental health intervention projects, (4) were based in the United States, (5) spoke English, and (6) were over 18 years of age.

### Ethical Considerations

This study was registered and classified as exempt by the Institutional Review Board of the University of California, Irvine (protocol 2761). All participants were provided with written informed consent via email prior to participating in the study and were invited via email to schedule an interview if they agreed to participate. Participants were informed that they could withdraw from the study at any time without penalty. Participants were compensated with a US $30 gift card after the interview. All data collected were deidentified before any analysis, and only the researchers assigned to this study had access to the data.

### Data Collection and Analysis

We started the semistructured interviews with participants after iterations of the interview protocol. We conducted 1 pilot interview. BO and KN conducted semistructured interviews via Zoom (Zoom Communications, Inc) that lasted 45 to 60 minutes. The interviews were conducted between May 2023 and February 2024. The authors, BO and KN, along with the participants, were present during the interviews. BO and KN were more junior than all participants (ie, a PhD candidate and a senior undergraduate student, respectively). BO and KN introduced themselves at the start of each interview, with one author conducting the interview, while the other turned off their camera and took notes. If the note-taking author had follow-up questions, they asked them at the end of the interview. All 12 interviews were video and audio-recorded with participants’ consent, transcribed, and analyzed. We transcribed all interviews using Otter.ai (Otter.ai, Inc), and the 2 interviewers reviewed and edited the transcripts for accuracy by comparing them to the recordings. The transcripts were not returned to participants for comment. Recruitment, data collection, and analysis were conducted concurrently until we reached saturation on the themes discussed in this study [43]. For instance, themes such as “engagement conceptualized as amount of usage” recurred frequently and were defined early in the interview process, with limited new insights emerging in later interviews. Other themes, such as “engagement that is not captured” (ie, invisible engagement) and “challenges to finding opportune moments for engagement,” appeared less frequently in our analysis but consistently recurred across participants with sufficient depth and consistency to support the development of these themes. By the end of the 12 interviews, we were no longer generating substantial new insights related to the study objectives.

We conducted a thematic analysis within a constructivist framework [44]. Thematic analysis involves the process of researchers familiarizing themselves with the data, generating initial codes, searching for themes, reviewing themes, defining and naming themes, and producing the report [44]. Two researchers used inductive coding to analyze the interviews [45] using the software ATLAS.ti (ATLAS.ti Scientific Software Development GmbH). First, each researcher familiarized themselves with the data and inductively coded 3 transcripts independently, and then compared interpretations [44]. We frequently iterated on the codes and discussed them with the entire research team as part of the thematic analysis process to make sense of the data and discuss potential themes that go beyond simple descriptions of the content [44,46]. Those codes were used to create a codebook, which included codes such as “experience creating digital tools,” “objective engagement,” and “intervention and technology together.” The 2 researchers continued to review codes weekly with each other and other team members until all transcripts had been reviewed. During the coding process, we started searching for themes [44]. We also took notes after interviews and wrote memos as we read the transcripts to discuss and resolve disagreements about codes and themes. The first iteration of affinity mapping to develop and review potential themes was done in person with physical sticky notes, which slowed the process and allowed the researchers to discuss the organization of codes and patterns [44,47]. Then, BO conducted pattern coding [45]. This process led to the conceptualization of themes such as “measurement of engagement” and “intervention vs technology.” As we produced this manuscript, we iterated on the themes. We did not share the data analysis and themes with participants.

## Results

### Participants

We interviewed 12 participants (5 men and 7 women). All participants were academic faculty. All participants had experience using participatory human-centered design (HCD) approaches and applied those approaches in their DMHI projects. Table 1 includes the background of participants, the focus of their DMHI, the modality of technology used for the DMHI (eg, SMS, app, and website), the development stage (prototype, pilot, or randomized controlled trial), and the target user group. A prototype refers to early versions of the DMHI, often cocreated with users; pilot refers to feasibility or pilot trials, during which the DMHI is refined; and randomized controlled trial refers to relatively advanced versions of the DMHI.

**Table 1.** Background of participants and overall goals of their DMHI^a^ projects.

ID	Background	DMHI project	Delivery modality	Development stage	Target user group
P1	Clinical psychology	Mental health symptom self-management and skill building	SMS	Pilot	Young adults
P2	Clinical psychology; Implementation science	Eating disorders, weight management, and skill building	App and SMS	Pilot	Adults with eating disorder
P3	Health communication	Mental health symptom self-management and skill building	SMS	Pilot	Young adults
P4	Social welfare	Mental illness self-management	App	Pilot	Adults diagnosed with serious mental illness
P5	Clinical psychology	Mental health counseling skills learning; depression and anxiety symptom management	Web and app	RCT^b^	Counseling trainees; people with depression and anxiety
P6	Experimental psychology	Mental health symptom self-management and skill building	Website	Prototype	Teenagers
P7	Primary care psychology	Mental health crisis prevention	SMS	RCT	Teenagers
P8	Psychiatry	Mental health crisis prevention	Web and SMS	Pilot	Teenagers with suicidal ideation
P9	Psychiatry	Depression assessment; symptom management and skill building	App	Pilot	Women in rural areas
P10	Clinical psychology	Mental health symptom self-management and skill building	Web, app, and SMS	RCT	People with depression and anxiety
P11	Clinical psychology	Mental health assessment and skill building tools for clients	Web	Pilot	Therapists
P12	Obstetrics	Mental health education and support; communication between providers and clients	App	Pilot	Pregnant people and new parents; clinical care managers

a DMHI: digital mental health intervention.

b RCT: randomized controlled trial.

### Overview

In this section, we first describe how clinical researchers operationalized engagement based on clinical outcomes but have started to include aspects of user experience. Then, we provide an overview of the engagement dimensions and specific findings for each of the dimensions. Finally, we present the barriers clinical researchers face in finding opportune moments for users to engage with DMHIs.

### Clinically Meaningful Outcomes Over Engagement With Technology

Participants highlighted that the clinical outcome, regardless of the amount or quality of user engagement with the technology, was their primary goal. In their pilots and clinical trials, engagement was not a main consideration in the design or evaluation of DMHIs. Instead, the focus was on the clinical outcomes because they are the traditional clinical research objectives and were also tied to the objectives outlined in their research grants. P5 stated, “Engagement was probably a secondary or tertiary consideration.” P5 explained that when considering engagement for DMHIs, “It was more about interface,” representing a common view among participants that engagement is tied primarily to the technology component of the DMHI.

Successful clinical outcomes meant achieving behavior change and clinically meaningful symptom reduction. Clinical researchers wanted users to sustain engagement with the learned behavior in their daily lives (ie, behavior change):

*It’s just putting those things into practice regularly over time....I think it was very eye opening...realizing what the heart of the problem was, that the technology solution needed to be...the sustaining behavior change piece*.

[P2]

Participants prioritized clinical outcomes over user engagement with the DMHI. Their goal was for users to achieve their mental health goals rather than to improve user engagement with the DMHI.

### Understanding of Engagement Evolved to Include User Experience

As their research projects progressed, participants’ understanding of engagement with DMHIs changed. At first, participants relied on their clinical expertise. Later, as participants worked more with HCI researchers and/or incorporated HCD methods (eg, co-design workshops and feedback sessions), they recognized the importance of the user experience in enhancing engagement with DMHIs. A participant reflected on their exclusive reliance on clinical experience and realized they needed to talk to users:

*I started with a needs assessment, and it changed the entire trajectory of my thinking on it. Now, it didn’t change clinically, the core principles we were going to be focusing on, but...how I was creating that into a package that would be really meaningful for people.*

[P2]

A participant said they had learned to prioritize user experience as well as adhere to psychotherapeutic principles:

*I’ve learned over time that...it matters a lot less how perfectly aligned to the psychotherapeutic concept something is....it has to be a real mix of what is actually engaging to somebody and what has sort of the spirit of the intervention built into it.*

[P1]

Participants said their understanding of engagement evolved to recognize the preferences and experiences of users:

*I think we have to change the way we think about* [engagement]. *It’s not just about like maximizing engagement, it’s about having enough there that people can find something useful.*

[P7]

*...But then making sure that the content we’re delivering is, you know, at the right reading level as the right kind of experience around it has the right images that might connect with participants.*

[P12]

As participants gained more experience in HCI and HCD approaches, their understanding of engagement evolved from focusing on clinical aspects, such as adherence, to including the user experience.

### Engagement Dimensions

We separate engagement, as described by participants, into different dimensions reflecting its complex nature based on our findings (Textbox 1). These dimensions include *DMHI components* (intervention, technology, and human support) [38,48], *levels of engagement* (micro and macro) [26,27], and *visibility of engagement* (visible and invisible).

Textbox 1. **Engagement dimensions for digital mental health interventions (DMHIs).**

**DMHI components**

- Intervention or intervention activities: engagement with the clinical or behavioral intervention, such as cognitive behavioral therapy, behavioral activation, and psychoeducation
- Technology: engagement with digital tools or platforms supporting the intervention, such as apps, websites, and text messages
- Human support: engagement with human support, such as behavioral coaches or therapists and/or technical support for the technology

**Levels of engagement**

- Micro level: moment-to-moment engagement with technology
- Macro level: engagement with intervention or intervention activities

**Visibility of engagement**

- Visible: engagement with any of the DMHI components that is visible to the researchers, either in person or digitally
- Invisible: engagement with any of the DMHI components that is not visible to the researchers

### Components for Engagement: Intervention, Technology, and Human Support

#### Overview

The 3 components of DMHIs that participants discussed were the intervention or the intervention activities, the technology, and the human support. Some participants viewed engagement with the intervention and with the technology as clearly separate, while many participants saw them as interconnected. Despite the differing views on engagement, participants wanted users to experience the DMHI as one cohesive intervention instead of separate components (eg, coach, technology, and behavioral activities).

#### Distinctions and Overlap of Intervention and Technology

Participants had different views about the DMHI’s intervention and technology components. Some participants saw the intervention and technology components as inseparable from each other. For example, a participant considered text messages (eg, SMS) sent to users as part of the intervention:

*It’s hard for me to differentiate them* [intervention and technology]*, because it’s a fully automated tool. So I do tend to think of the text messages as the intervention...*

[P3]

Some participants found it difficult to separate the technology and the intervention because of a perceived overlap between components. A participant explained that some therapist-related activities, such as seeing assessment results, could be seen as part of the technology because (1) they were done within the technology and (2) they were not part of the *key* components of the intervention for that clinical trial. At the same time, those activities were part of *every* intervention:

*...Another example of engaging with the app, but then not really [with] the intervention components is looking at the review screen and seeing your clients. But that’s an important part of every intervention is looking at your clients progress over time and assessment results in our completion*.

[P11]

On the other hand, some participants viewed technology as a medium (eg, SMS) for an intervention (eg, message content). While the core of the intervention remained consistent, its delivery could be adapted across different technologies, such as a website or mobile app. In that case, the design of the technology could be changed to enhance how users engage with the intervention. A participant provided an example of a digital tool that asked users to write text messages:

*[the technology choice] changes how somebody relates to the tool, or how somebody is actually engaging, the physical difficulty of writing detailed and long text messages. And the annoyance factor of it is real*.

[P1]

Another reason that participants viewed the technology as mostly separate was that the intervention encompassed psychotherapy activities to be completed outside of the technology:

*A lot of CBT also requires for folks to continue to do that skill throughout the week, in the mindset of like a weekly psychotherapy session... is not the same as logging onto the app and tracking somethin*g.

[P5]

Some participants emphasized the need to differentiate and unpack the differences between the intervention and the technology to understand how to best operationalize engagement:

*I do think that they kind of like are overlapping, like in a Venn diagram, right... I think I would more carefully look at unpacking, engagement with the intervention versus engagement with the technology as a way to connect A to B*.

[P5]

Although some participants viewed the intervention and technology as separate when operationalizing engagement, they said it was important for users to feel the intervention and technology as one for the purpose of creating a seamless user experience:

*Ideally, from the perspective of the patient, and the adults supporting that patient, there should be no obvious difference between the different parts of the intervention, it should all feel like the same thing to them*.

[P8]

In summary, participants had different perspectives on whether the intervention and technology were inseparable (eg, text messages that constitute the intervention but require the technology) or separable (eg, the technology serves as the medium through which the intervention is delivered). Some participants discussed how users should experience the technology and intervention as one, but noted that it is useful for operationalization to clearly separate both components.

#### Human Support

Participants argued that human support, such as coaches and therapists, can help users understand and engage with a DMHI. Participants recognized that human support is considered a separate component from the technology and the intervention. They explained that coaches are not necessarily therapists but are individuals trained to provide personalized feedback, guidance, and encouragement. Coaches provide a human presence for accountability, personalized feedback, and encouragement to users [49]. Many participants included human support in the pilots and clinical trials of their DMHIs:

*...We’re running a trial right now to see if coaching might add value to the intervention. In that context, the coach is also a component of the intervention. That’s not the technology. So, I guess I think of the intervention as the technology plus the support and context for technology use*.

[P3]

Human support was seen as essential to fostering engagement, although it was not always present in DMHIs. It was discussed as a designed component of DMHIs, distinct from both the intervention and the technology.

### Engagement Levels: Balancing Micro- and Macro-Approaches

Participants discussed concepts aligned with the micro- and macroengagement levels proposed by Yardley et al [26] (Textbox 1). However, they did not use this terminology; instead, they discussed users’ moment-to-moment interactions with the DMHI itself or users’ involvement with the intervention processes and goals. Participants measured engagement success in terms of clinically meaningful outcomes, which happen through macroengagement (eg, practicing therapeutic skills in and outside the app). However, many of the measurement tools primarily measured engagement at the micro level (eg, logging into the app). All participants used quantitative metrics to evaluate their DMHI, with some also incorporating qualitative methods. Quantitative measures of engagement traditionally focused on usage data (eg, number of clicks or the amount of usage of different features):

*How many contact points do they have or touch points? And how deeply engaged are they by like, using all the different features of it?*

[P1]

Participants emphasized that clinically meaningful outcomes required more macroengagement (ie, engagement with the intervention goals) and not necessarily frequent or prolonged engagement with the technology. One participant explained:

*...ideally, what you would hope to see, in order to see an effect, is that people are trying something. So, not just learning about it, but taking a step in their daily life*.

[P3]

Given participants’ focus on macroengagement as a way to achieve meaningful clinical outcomes, some participants expressed skepticism about the ability of quantitative metrics alone to fully capture whether users are engaging with the intervention’s goals:

*Just because a person’s clicking around does not necessarily mean that they’re utilizing it in an effective way*.

[P2]

Some participants discussed the need for a shift toward using more qualitative approaches (eg, user satisfaction) to complement the understanding of engagement. To gain deeper insights into engagement, especially macroengagement, a participant said that it is important to understand how users think and feel about the DMHI:

*What if they’re responding [to the intervention], but they’re quite dissatisfied? Is that engagement that we would want to see? Maybe not? So, you know, we’ve tried to look, we’ve tried to ask about, how do you feel about the program? What’s the kind of relationship that’s built with the program? Do you think about it, do you... So kind of getting a broader sense of what it means to people*.

[P3]

Another perspective on engagement levels was that even a little microengagement could lead to a successful engagement. Some participants considered a successful engagement with DMHIs to be any progress that users made toward their goals (eg, behavior change and symptom reduction), even if the users had not yet achieved their main mental health goal (ie, intervention outcome). A participant stated that even a little engagement with the DMHI (ie, microengagement) could propel someone to get better when they are in their worst mental health state:

*People often go and start using digital mental health tools, or any treatment, when they’re at their worst....that’s when you go in and you say “I'm done with this.” And often just the decision to start doing that is kind of a sign that the person is starting to want to improve and take steps*.

[P10]

That perspective highlights how a quantitative-only approach would be insufficient to understand user engagement with the DMHI because it misses the qualitative aspects (eg, user satisfaction, feelings, and relationship with the DMHI) that lead users toward macroengagement.

### Engagement Visibility

#### Overview

Participants wanted to understand both the users’ visible and invisible engagement. Visible engagement refers to the aspects of engagement that can be captured by researchers. This is usually done using quantitative metrics (eg, how often users opened the app), but it can also include qualitative data (eg, how users felt about using the app). On the other hand, contextual issues such as individual barriers and challenges are often invisible to researchers. This could also include how users engaged with the intervention. Participants said one of their main challenges is accounting for those invisible factors during users’ engagement with a DMHI. Because those factors that affect engagement (eg, environment, thoughts, feelings, and attitudes) are not visible to researchers, we label them as “invisible engagement.”

#### Visible Engagement as Measurable Interactions

Participants primarily used quantitative metrics as indicators of engagement, such as user interaction levels. In one participant’s study, users were required to complete a specific percentage of the interactions. Another participant conceptualized engagement based on different measurable interactions with the app, such as the number of sign-ups, how often users log in, and whether therapists created assessments to be used in the app:

*...How, at the very top level is, are we looking at signups?...how often are the therapists logging in, over the course of time?...That’s on the high level, but then on a lower, how many assessments are therapists assigning?...*

[P11]

Visible engagement refers to the user interactions that are more easily observable and measurable by researchers. Researchers primarily use quantitative metrics to capture these user interactions with the DMHI.

#### Invisible Engagement as Interactions That Are Not Captured

Participants consistently highlighted that users encountered barriers in their lives that prevented them from engaging with the DMHI. Participants said that users were often busy, overwhelmed, or lacked resources. A participant noted that DMHIs needed to fit into people’s daily life contexts:

*I think increasingly, in terms of the patient engagement, ...how to fit it into the fabric of people’s lives, you know, where are the touch points, sort of understanding kind of, how do you, if it’s on a phone, when are they looking at their phone? What’s the context in which they’re willing to do these things?*

[P10]

Participants said that it was difficult to align the DMHI to people’s various daily schedules because they could not capture the users’ environments and behaviors outside of the research environment. These characteristics were invisible to them and, therefore, could not be easily captured by current methods. A participant compared the engagement with the intervention that was not tracked through the technology (ie, invisible engagement) to a black box that the researcher did not have access to:

*And then engagement, I guess was, to me, like the black box that happens when they go home to do it. Are they doing it? For how long? Did they get bored? Did they finish?*

[P5]

The “black box” analogy given by P5 highlights the challenges of capturing invisible engagement activities. It was difficult to capture context, behaviors, thoughts, and feelings at the time of engagement when they were outside of the research environment. Those aspects usually encompassed users’ challenges in capturing invisible engagement.

Capturing the invisible engagement was important to understand how engagement is happening with the intervention, but that was challenging to do:

*I think that’s often kind of a challenge within the design of the interventions to understand what the person is doing. Because much of the intervention part, hopefully, if it’s effective, they’re going to be doing outside of the app or the device, and we’re not going to have any visibility into it*.

[P10]

Participants tried to make invisible engagement more visible through qualitative questionnaires or behavior change assessments. For example, a participant sent questions to users, asking if they engaged in the activities they were supposed to:

*There’s a lot of invisible behavior... So, at least once a day, people are asked questions “were you able to do a positive activity today? Reply with yes or no.”...Even though they’re not required or incentivized to respond, it is one way that we are able to see if people are staying engaged*.

[P3]

However, those questionnaires and assessments only provide a partial view of invisible engagement, as they are unable to capture the complexity of users’ daily life contexts:

*We don’t have that much insight into kind of the context in which people are using the program*.

[P3]

Moreover, a major challenge to including qualitative approaches in research was the funding mechanisms that prioritized clinical adherence, clinical outcomes, and quantitative measures:

*I think the former* [quantitative metrics] *is, unfortunately, what kind of feel bound to with grants and papers*.

[P6]

Participants tried to capture aspects of invisible engagement through qualitative questionnaires and assessments. However, those approaches were often insufficient to get a complete view of the users and their context. Furthermore, prioritizing quantitative methods made it difficult to use qualitative approaches that might be helpful in this regard.

### Challenges in Finding Opportune Moments for Engagement

Participants said it was challenging to match the delivery time of a DMHI with opportune moments for engagement. For example, a DMHI involved text messages that users needed to reply to so that engagement would happen. In that case, *opportune moments* were the moments when users were available to reflect on and reply to the text message. The participants noticed that they had better response rates when the time at which the intervention was sent intersected with an opportune moment for the users to answer:

*...We have seen sometimes a text message will intersect with these opportune moments. And catching people during those is extremely effective and can help continue that. If the metric is like, number of days the tool has been responded to, for example, we’ll see pretty good engagement on that*.

[P1]

Some participants asked users to select their preferred time for receiving messages, aiming to enhance engagement at opportune moments:

*We are offering people the opportunity to select what time they received it. That was also partially around convenience, but also around engagement. Because we thought if they picked it, then maybe they would be more likely to interact*.

[P7]

However, asking users for their preferred time to receive reminders did not completely solve the problem of reaching users at the right time. To address this, a participant was exploring ways to reach users at opportune moments for engagement, based on user needs and context, but they had not yet found a good solution:

*We’re building tools to try to be able to figure that out* [what are the opportune moments]. *But we don’t totally know... based on the data we’re collecting, how to figure out for a particular person what is the right time*.

[P1]

Participants highlighted that delivering the intervention when users were available and willing to engage improved response rates and engagement. However, understanding each user’s opportune moments for engagement, which are often invisible, remained a major challenge.

## Discussion

### Principal Findings

Clinical mental health researchers who created DMHIs navigated tensions between the complexity of engagement dimensions, users’ uncaptured experiences, and their project constraints. Participants focused on measuring microengagement activities, but their project goals were tied to macroengagement activities. This issue highlights one of the main challenges in clearly operationalizing engagement for DMHIs—the disconnect between what is being measured and the goals of the DMHI, which reflects the broader lack of consensus on how engagement should be operationalized. In this section, we discuss the challenges of designing for engagement and suggest design considerations based on participants’ research practices and barriers. Our findings unpack engagement into dimensions that can guide the choice of engagement strategies and design decisions for DMHI projects.

### Designing for Engagement

DMHI researchers faced a tension between measuring clinical outcomes and understanding user engagement. One of the key differences between clinical outcomes and user engagement is that clinical outcomes are measured by *end point* results, captured in the *controlled environment* of a clinical trial, whereas user engagement is a dynamic *process* of users’ interactions with the DMHI and is best understood in *real-world* settings. Researchers primarily captured engagement at the micro level, which provided a snapshot of users’ moment-by-moment interactions with the technology. However, this often failed to capture the contextual and longitudinal complexities of engagement [10,26,50] with DMHIs. This issue can be addressed by incorporating a macro-level perspective (ie, users’ engagement with the broader intervention goals) that includes longitudinal user experiences, context, and changes over time [26,27,50,51]. Focusing on macro-level engagement could help in understanding the dynamic nature of mental health in relation to longitudinal engagement with the DMHI.

The tension between the focus on clinical outcomes and users’ experiences leads to different understandings of “successful engagement.” Due to the focus on clinical outcomes, clinical research frameworks often overlook some of the benefits that users gain from DMHIs. In contrast, some participants considered an intervention successfully engaging for users even when it led to small benefits (eg, taking a first step for behavior change), regardless of whether the intervention achieved the intended outcome. This perspective shifts the understanding of “successful engagement” from solely focusing on clinical outcomes to also recognizing small user progress and subjective benefits, which aligns with HCD and HCI literature [18]. Prior research indicates that understanding how users engage with a DMHI (eg, how much control they thought the DMHI gave them over activities in their lives; how easy they thought it was to use the DMHI) can provide better insights into the mechanisms of change of a DMHI rather than just quantitative usage metrics. This is because usage data were found not to be significantly related to clinical outcomes [52]. That highlights the importance of using a mix of quantitative and qualitative data to capture how users engage, the user experience, and the clinical outcomes in both clinical research and real-world settings. However, despite recognizing the value of qualitative approaches, our findings show that participants found it difficult to prioritize those methods in clinical research settings due to the need for more time and resources (eg, researchers and funding).

The dimensions of engagement (Textbox 1) can help researchers better prioritize aspects of engagement that are important to them when designing DMHIs. Researchers can examine individual dimensions (technology, intervention, and human support components; micro- and macro-levels of engagement; and visible and invisible engagement) to determine which one(s) may need to be addressed to help them reach their design goals. For example, at the component level, the technology may need to be adapted for users unfamiliar with digital systems, or an intervention may need to include terminology that is culturally appropriate [53,54]. Similarly, if the goal is for users to apply therapeutic skills in their daily lives (ie, macroengagement), researchers should prioritize understanding users’ contexts and behaviors outside the app rather than focusing only on in-app interactions. At the same time, while focusing on individual dimensions to understand their impact, it is just as important to understand how these dimensions affect one another. Overlooking these interdependencies can make a design decision made for one component unintentionally hinder engagement in another. For example, in an area with poor internet service, the need for a lightweight website (ie, technology) affects the design of the content (ie, intervention). Consequently, we suggest that researchers explicitly map, early on, which dimensions and interdependencies are most important for their DMHI. These decisions will affect resource allocation and the choice of relevant design and measurement approaches.

### Visible vs Invisible Engagement

Participants reported that one of their main challenges was capturing users’ changing behavior and context when engaging with the DMHI. Based on our findings, *visible engagement* refers to the user engagement that researchers can measure, such as engagement conducted digitally and captured by log data, or that happens in person in a setting (eg, a hospital) where researchers can observe these activities. This includes usage data in an app or interactions with a coach. In contrast, *invisible engagement* (Table 2) refers to user engagement that is not directly observable and not easily measured, such as when users practice their target behavior outside the DMHI app or intervention environment. However, invisible engagement can become visible if it is captured; for example, if researchers are able to track that a person is working on the intervention activity outside of the app.

**Table 2.** Example of visible and invisible engagement for DMHI^a^ components.

	Visible engagement	Invisible engagement
Intervention or target behavior	Watching videos on the technology tool; using the app every week; going through a behavioral activity with the coach	Practicing behavioral activities outside DMHI technology (eg, offline and other apps); reflecting on an activity
Technology	Downloading the app; clicking through the technology tool	Feeling connected (ie, affect) to the technology tool
Human support	Calling a coach; taking notes from the call	Thoughts (ie, cognition) and feelings (ie, affect) during a call with a coach

a DMHI: digital mental health intervention.

Capturing invisible engagement could help identify opportune moments of engagement, thereby increasing user response rates and engagement with the DMHI, which could improve clinical outcomes. Currently, participants mostly rely on users to indicate to them when it would be a good time to receive an intervention activity, but barriers can unexpectedly hinder engagement. By capturing invisible engagement, researchers could understand contextual triggers, such as users’ emotional states, environments, and times that support or hinder engagement, and deliver future interventions at moments when users are more likely to engage.

Therefore, the best approach for researchers aiming to capture invisible engagement with a DMHI is through a combination of methods. Participants used methods such as ecological momentary assessments and questionnaires. However, participants stated that these could lead to inaccurate insights into invisible engagement because they rely on the user’s memory and self-report. Ecological momentary assessments have the benefit of assessing longitudinal experiences and reducing recall bias, but they still rely on self-report and may be burdensome to users [55]. Passive sensing methods to collect information on phone usage patterns, activities, or location can offer researchers a way to capture physiological data and more contextual information without increasing the burden on users. Qualitative approaches could add depth to the understanding of the different types of engagement. For example, microphenomenological interviews, which focus on interviewing and analyzing a specific subjective experience [56], could yield insights into cognitive and affective engagement that can be harder for users to explain without this specific probing. Capturing more information from some users about what is usually invisible gives more researchers greater insights into how to continue iterating on the design to improve and maintain engagement after the DMHI is implemented.

Nonetheless, several aspects of invisible engagement can be difficult to make visible with current approaches. Technical, administrative, and ethical barriers, such as sensors breaking, which results in incomplete or unreliable data, data breaches and privacy concerns [26,57], and limited resources to apply additional methods, limit what is feasible to capture. The goal should be to make selective aspects of engagement visible, specifically the ones that researchers hypothesize to affect outcomes and user experience, rather than trying to capture all aspects of invisible engagement.

Through our data analysis, we have identified 2 different dimensions of engagement. First, as described in the Results section, we found that engagement occurred at both the macro- and micro-levels. Yardley et al [26] have called for researchers to operationalize engagement for digital behavior change interventions at the micro- and macro-levels. We add to this understanding by illustrating how these dimensions are reflected in digital mental health interventions, focusing macro-level engagement on activities related to behavior change (ie, using therapeutic skills during the week without tracking them in the app) and the micro-level engagement on activities related to technology use (ie, clicking on features in the app).

Our analysis highlights that participants viewed microengagement as interactions that are more readily captured and, therefore, visible, whereas macroengagement was more difficult to capture and, therefore, often invisible. However, the relationship between these 2 different dimensions of engagement may not be a simple 1:1 correspondence, as suggested by our participants. Future research should further examine these relationships across different intervention contexts.

Future work should investigate the process of how engagement occurs during specific moments of interaction with a DMHI, including invisible engagement, to inform the design of systems that enhance engagement. This could include studies with potential users of DMHIs, examining the cognitive, affective, and behavioral constructs of engagement within each dimension, as well as the interactions between dimensions.

### Limitations

This study has a few limitations. As a qualitative study, the findings may not be generalizable to all researchers creating and implementing DMHIs. Moreover, all participants were academic researchers in the United States. Academic institutions in the United States face specific academic constraints and operational requirements tied to grant funding and clinical trials, which could affect how engagement is operationalized. Specifically, these constraints can limit the time or financial resources available to prioritize engagement during project design. Therefore, the results may not represent industry settings or cultural contexts outside of the United States. BO and KN conducted the interviews together. The presence of 2 interviewers may have introduced social desirability bias. To partially mitigate that bias, the researcher who was not interviewing turned off their camera and microphone after introducing themselves and took notes until close to the end of the interview. Finally, the interviews were mostly conducted after the engagement aspects of the projects had concluded, so the findings were based on recall. We did not use any mitigation strategies for recall bias, such as reviewing project documents or sending follow-up emails. Consequently, participants may have omitted details or misremembered aspects of their projects during the interview.

### Conclusions

This study contributed to the understanding of engagement in DMHIs. Building on prior conceptualization, we proposed that 3 dimensions of engagement (ie, components, levels, and visibility) can guide more intentional design for DMHIs and other health-related technologies. By introducing visibility as a dimension of engagement, our work highlights aspects of engagement that occur beyond what researchers can readily observe and measure. Specifically, this study highlighted a tension between clinical mental health researchers’ goals and the qualitative and experiential aspects of engagement that are often not captured in DMHIs. Researchers created DMHIs with macroengagement as a goal, but mainly captured microengagement through quantitative usage metrics and end-point results, missing the qualitative dynamics of user engagement in real-world settings. Making visibility explicit may support researchers in accounting for aspects of engagement that matter most for their research goals but are harder to capture.

## Abbreviations

DMHI: digital mental health intervention
HCD: human-centered design
HCI: human-computer interaction

## References

[1] Wright E, Dore EC, Koenen KC, Mangurian C, Williams DR, Hamad R (2025). Prevalence of and inequities in poor mental health across 3 US surveys, 2011 to 2022. JAMA Netw Open.

[2] Mohr DC, Ho J, Duffecy J (2010). Perceived barriers to psychological treatments and their relationship to depression. J Clin Psychol.

[3] Lyon A, Munson SA, Reddy M (2023). Bridging HCI and implementation science for innovation adoption and public health impact. Ext Abstr Hum Factors Computing Syst.

[4] Linardon J, Cuijpers P, Carlbring P, Messer M, Fuller-Tyszkiewicz M (2019). The efficacy of app-supported smartphone interventions for mental health problems: a meta-analysis of randomized controlled trials. World Psychiatry.

[5] Weisel KK, Fuhrmann LM, Berking M, Baumeister H, Cuijpers P, Ebert DD (2019). Standalone smartphone apps for mental health: a systematic review and meta-analysis. NPJ Digit Med.

[6] Edge D, Watkins ER, Limond J, Mugadza J (2023). The efficacy of self-guided internet and mobile-based interventions for preventing anxiety and depression: a systematic review and meta-analysis. Behav Res Ther.

[7] Lattie EG, Adkins EC, Winquist N, Stiles-Shields C, Wafford QE, Graham AK (2019). Digital mental health interventions for depression, anxiety, and enhancement of psychological well-being among college students: systematic review. J Med Internet Res.

[8] (2021). Comprehensive mental health action plan 2013-2030. https://iris.who.int/server/api/core/bitstreams/69921758-6229-49ba-bd3d-c24736e35829/content.

[9] Donkin L, Christensen H, Naismith SL, Neal B, Hickie IB, Glozier N (2011). A systematic review of the impact of adherence on the effectiveness of e-therapies. J Med Internet Res.

[10] Doherty K, Doherty G (2019). Engagement in HCI: conception, theory and measurement. ACM Comput Surv.

[11] Arnold C, Williams A, Thomas N (2020). Engaging with a web-based psychosocial intervention for psychosis: qualitative study of user experiences. JMIR Ment Health.

[12] Ben-Zeev D, Brian RM, Jonathan G (2018). Mobile health (mHealth) versus clinic-based group intervention for people with serious mental illness: a randomized controlled trial. Psychiatr Serv.

[13] Torous J, Michalak EE, O’Brien HL (2020). Digital health and engagement-looking behind the measures and methods. JAMA Netw Open.

[14] Ng MM, Firth J, Minen M, Torous J (2019). User engagement in mental health apps: a review of measurement, reporting, and validity. Psychiatr Serv.

[15] Flett JAM, Hayne H, Riordan BC, Thompson LM, Conner TS (2019). Mobile mindfulness meditation: a randomised controlled trial of the effect of two popular apps on mental health. Mindfulness (N Y).

[16] Baumel A, Muench F, Edan S, Kane JM (2019). Objective user engagement with mental health apps: systematic search and panel-based usage analysis. J Med Internet Res.

[17] Lipschitz JM, Pike CK, Hogan TP, Murphy SA, Burdick KE (2023). The engagement problem: a review of engagement with digital mental health interventions and recommendations for a path forward. Curr Treat Options Psychiatry.

[18] Perski O, Blandford A, West R, Michie S (2017). Conceptualising engagement with digital behaviour change interventions: a systematic review using principles from critical interpretive synthesis. Transl Behav Med.

[19] (1999). Bridging science and service: a report by the national advisory mental health council’s clinical treatment and services research workgroup. National Institute of Mental Health (NIMH).

[20] Kazantzis N, Whittington C, Dattilio F (2010). Meta-analysis of homework effects in cognitive and behavioral therapy: a replication and extension. Clin Psychol Sci Pract.

[21] Chorpita BF, Daleiden EL, Weisz JR (2005). Identifying and selecting the common elements of evidence based interventions: a distillation and matching model. Ment Health Serv Res.

[22] Kelders SM, Kip H, Beerlage-de Jong N, Köhle N (2024). What does it mean to be engaged with digital health interventions? A qualitative study into the experiences of engaged users and the views of professionals. Digit Health.

[23] Kelders SM, van Zyl LE, Ludden GDS (2020). The concept and components of engagement in different domains applied to eHealth: a systematic scoping review. Front Psychol.

[24] O’Brien HL, Toms EG (2008). What is user engagement? A conceptual framework for defining user engagement with technology. J Am Soc Inf Sci.

[25] Nahum-Shani I, Yoon C (2024). Towards the science of engagement with digital interventions. Curr Dir Psychol Sci.

[26] Yardley L, Spring BJ, Riper H (2016). Understanding and promoting effective engagement with digital behavior change interventions. Am J Prev Med.

[27] Cole-Lewis H, Ezeanochie N, Turgiss J (2019). Understanding health behavior technology engagement: pathway to measuring digital behavior change interventions. JMIR Form Res.

[28] Yeager CM, Benight CC (2022). Engagement, predictors, and outcomes of a trauma recovery digital mental health intervention: longitudinal study. JMIR Ment Health.

[29] Zech JM, Johnson M, Pullmann MD (2023). An integrative engagement model of digital psychotherapy: exploratory focus group findings. JMIR Form Res.

[30] Cho HN, Eisenberg D, King C, Zheng K (2026). EngageTriBoost: predictive modeling of user engagement in digital mental health intervention using explainable machine learning. arXiv.

[31] Kelders SM, Kip H, Greeff J (2020). Psychometric evaluation of the TWente Engagement with Ehealth Technologies Scale (TWEETS): evaluation study. J Med Internet Res.

[32] O’Brien HL, Cairns P, Hall M (2018). A practical approach to measuring user engagement with the refined user engagement scale (UES) and new UES short form. Int J Hum Comput Stud.

[33] Jansen BJ, Guan KW, Salminen J, Aldous KK, Jung SG (2025). What is user engagement?: a systematic review of 241 research articles in human-computer interaction and beyond. Proc 2025 CHI Conf Hum Factors Comput Syst.

[34] O’Brien H, Filimowicz M, Tzankova V (2018). New Directions in Third Wave Human-Computer Interaction: Volume 2 - Methodologies.

[35] Kornfield R, Zhang R, Nicholas J (2020). “Energy is a finite resource”: designing technology to support individuals across fluctuating symptoms of depression. Proc SIGCHI Conf Hum Factor Comput Syst.

[36] Cavanagh K, Millings A (2013). Increasing engagement with computerised cognitive behavioural therapies. ICST Trans Amb Sys.

[37] Bunnell BE, Nemeth LS, Lenert LA (2021). Barriers associated with the implementation of homework in youth mental health treatment and potential mobile health solutions. Cogn Ther Res.

[38] Mohr DC, Lyon AR, Lattie EG, Reddy M, Schueller SM (2017). Accelerating digital mental health research from early design and creation to successful implementation and sustainment. J Med Internet Res.

[39] Morrison LG, Yardley L, Powell J, Michie S (2012). What design features are used in effective e-health interventions? A review using techniques from critical interpretive synthesis. Telemed J E Health.

[40] Richards D, Richardson T (2012). Computer-based psychological treatments for depression: a systematic review and meta-analysis. Clin Psychol Rev.

[41] Gilbody S, Littlewood E, Hewitt C (2015). Computerised cognitive behaviour therapy (cCBT) as treatment for depression in primary care (REEACT trial): large scale pragmatic randomised controlled trial. BMJ.

[42] Fink A (2017). How to Conduct Surveys: A Step-by-Step Guide.

[43] Hennink MM, Kaiser BN, Marconi VC (2017). Code saturation versus meaning saturation: how many interviews are enough?. Qual Health Res.

[44] Braun V, Clarke V (2006). Using thematic analysis in psychology. Qual Res Psychol.

[45] Saldaña J (2025). The Coding Manual for Qualitative Researchers.

[46] Hill CE, Thompson BJ, Williams EN (1997). A guide to conducting consensual qualitative research. Couns Psychol.

[47] Maher C, Hadfield M, Hutchings M, de Eyto A (2018). Ensuring rigor in qualitative data analysis: a design research approach to coding combining NVivo with traditional material methods. Int J Qual Methods.

[48] Schueller SM, Torous J (2020). Scaling evidence-based treatments through digital mental health. Am Psychol.

[49] Mohr DC, Cuijpers P, Lehman K (2011). Supportive accountability: a model for providing human support to enhance adherence to eHealth interventions. J Med Internet Res.

[50] Villegas Mejía C, Remmerswaal D, Engels RCME, Ludden GDS, Boffo M (2024). Macro-engagement in mHealth: exploring user engagement beyond the screen. Digit Health.

[51] O’Brien HL, Morton E, Kampen A, Barnes SJ, Michalak EE (2020). Beyond clicks and downloads: a call for a more comprehensive approach to measuring mobile-health app engagement. BJPsych Open.

[52] Graham AK, Kwasny MJ, Lattie EG (2021). Targeting subjective engagement in experimental therapeutics for digital mental health interventions. Internet Interv.

[53] Cheng NY, Wong N, Reddy M (2025). Understanding mental wellbeing and tools for support with Taiwanese emerging adults: an eastern cultural perspective. Proc 2025 CHI Conf Hum Factors Comput Syst.

[54] Bautista JR, Wong N, Reddy M, Schueller SM (2025). Healing through stories: co-designing digital mental health with Asian Americans. Proc 2025 CHI Conf Hum Factors Comput Syst New York.

[55] Doherty K, Balaskas A, Doherty G (2020). The design of ecological momentary assessment technologies. Interact Comput.

[56] Heimann K, Nouwens M, Saggurthi S, Dalsgaard P (2025). Micro-phenomenology as a method for studying user experience in human-computer interaction. Proc 2025 CHI Conf Hum Factors Comput Syst.

[57] González-Pérez A, Matey-Sanz M, Granell C, Díaz-Sanahuja L, Bretón-López J, Casteleyn S (2023). AwarNS: a framework for developing context-aware reactive mobile applications for health and mental health. J Biomed Inform.

